# Reduced Expression of Argonaute 1, Argonaute 2, and TRBP Changes Levels and Intracellular Distribution of RNAi Factors

**DOI:** 10.1038/srep12855

**Published:** 2015-08-05

**Authors:** Masayuki Matsui, Liande Li, Bethany A. Janowski, David R. Corey

**Affiliations:** 1 Departments of Pharmacology and Biochemistry, University of Texas Southwestern Medical Center, Dallas, Texas, 75390-9041.

## Abstract

Until recently, Argonaute 2 (AGO2) and other RNA factors were believed to be restricted to the cytoplasm of mammalian somatic cells. It is now becoming appreciated that RNAi factors can also be found in cell nuclei, but much remains to be learned about their transport, molecular recognition, and function. We find that siRNA-mediated reduction of AGO1 or AGO2 increases the proportion of AGO1 or AGO2 in cell nuclei. Inhibition of AGO1 expression led to increased AGO2 levels, while knockdown of AGO2 led to increased levels of AGO1. Blocking AGO1, AGO2, or TRBP expression changed expression levels and nuclear distribution of RNAi factors Dicer, TNRC6A (GW182), and TRBP. These data reveal the expression of RNAi proteins is mutually dependent and that perturbation can affect subcellular distribution of those factors inside cells.

The control of gene expression by RNA interference (RNAi) is often assumed to be restricted to the cytoplasm of mammalian somatic cells. Over the past decade, however, reports have implicated small RNAs and protein RNAi factors in the control of splicing^1^ and transcription^2^,^3^. These reports raise the possibility that RNAi, already known to be a powerful regulatory system in the cytoplasm, might also have the potential to be broadly active in nuclei.

Two potential mechanisms have been described to explain the control of splicing by the RNAi machinery. In one mechanism, binding of duplex RNAs targeting exonic or intronic sequences close to an exon increases histone H3 lysine 9 dimethylation and other heterochromatin marks to affect RNA polymerase processivity and modify the outcome of alternative splicing^4^,^5^. In another mechanism, our laboratory has demonstrated that duplex RNAs complementary to aberrant splice sites within exons or introns can affect alternative splicing of the disease genes dystrophin or SMN2 in an AGO2-dependent manner^6^. The mechanism we observe involves direct binding of RNA to pre-mRNA.

Effects of small RNAs on silencing transcription were first noted by Morris and colleagues in 2004^7^. Since that time reports have appeared implicating small RNAs as transcriptional activators^8^,^9^,^10^ and providing further support for transcriptional gene silencing^11^,^12^,^13^. Multiple mechanisms may be involved in RNA-mediated transcriptional regulation and important details remain unresolved. Studies concur, however, that RNA-mediated gene regulation requires Argonaute (AGO) protein^14^,^15^,^16^ and that recognition of small RNAs occurs at nascent transcripts that overlap the target gene, not at chromosomal DNA^17^,^18^,^19^,^20^. Once RNA-AGO complexes are bound to nascent RNAs, they act in cis to modulate transcription factor binding, histone modification, and RNA polymerase recruitment at the gene promoter. miRNAs have been observed to participate both in transcriptional gene silencing^21^,^22^ and activation^19^, suggesting a previously unanticipated potential for nuclear RNA-mediated mechanisms to control endogenous gene expression.

Given the potential for nuclear RNAi to have a profound effect on gene regulation and reports of nuclear RNAi in other classes of organism^23^,^24^,^25^, it may appear surprising that the phenomenon has not been explored more widely in mammalian cells. One explanation has been widespread skepticism that AGO and other RNAi factors were present in cell nuclei^26^,^27^ dating from the earliest period of research into mammalian RNAi.

AGO2 is well-known as the catalytic engine driving cytoplasmic RNAi^28^,^29^ and knowledge of AGO2 in mammalian cell nuclei has been limited. Several reports have described the presence of RNAi factors inside mammalian cell nuclei^30^,^31^,^32^ consistent with the observations that RNA and RNAi factors influence classical nuclear processes such as splicing and transcription. Because of uncertainty about the presence of RNAi factors within nuclei and technical difficulties performing definitive studies, many of the molecular details behind nuclear functions of RNAi factors have remain unresolved^33^.

Here we examine levels and subcellular distributions of two AGO variants, AGO1 and AGO2, and other RNAi factors, when cellular concentrations of AGO1/AGO2 are reduced using siRNAs in T47D and MDA-MB-453 cells. Our data reveal that each AGO variant is retained in the cell nuclei relative to the cytoplasm after the depletion of each AGO, while cellular levels of AGO1/AGO2 are regulated in a compensatory manner. These data have implications for experiments investigating functions of nuclear AGO proteins and suggest that cells may have mechanisms that maintain pools of nuclear AGO proteins.

## Results

### Isolation of nuclear extracts after transfection with anti-AGO siRNAs

**Figure 1:**
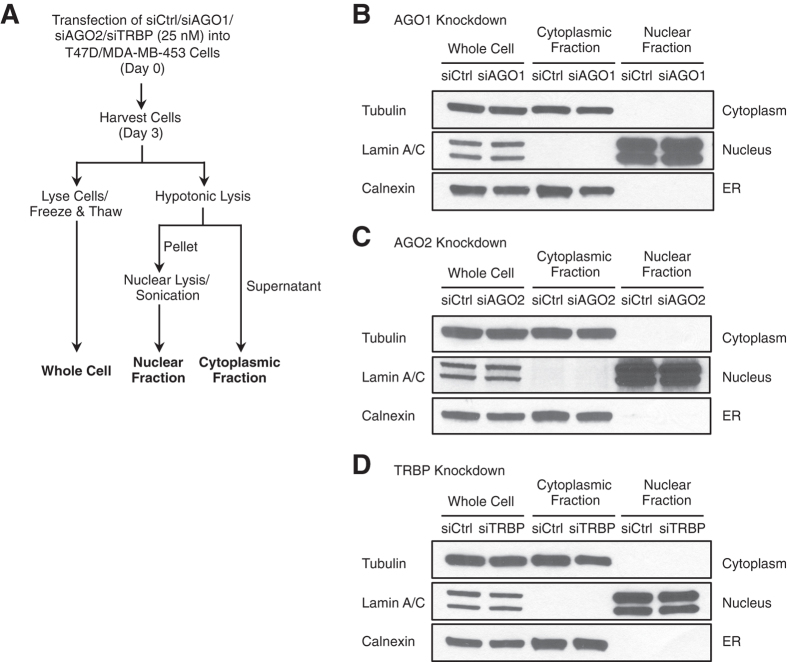
Subcellular fractionation of T47D cells to isolate whole cells, cytoplasmic fractions, and nuclear fractions. (**A**) Scheme of dsRNA transfection and subsequent subcellular fractionation. siRNAs specific for AGO1, AGO2, or TRBP and control dsRNA (siCtrl) were transfected into cells at 25 nM using Lipofectamine RNAiMAX. (**B**–**D**) Western blot analysis for Tubulin (cytoplasmic marker), Lamin A/C (nuclear marker), and Calnexin (ER marker) showing the purity of each fraction prepared from siCtrl-, siAGO1-, siAGO2, or siTRBP-treated T47D cells. Equal amount of proteins (25 μg) from each fraction were analyzed by SDS-PAGE. Similar western blot data for MDA-MB-453 cells are presented in Figure S1.

Accurate investigation into the presence of AGO in human cell nuclei depends on stringent purification to exclude cytoplasmic AGO. This is not a trivial task because AGO can be found in the endoplasmic reticulum (ER)^34^, an organelle closely associated with the nuclear membrane. We used optimized isolation procedures for this study to separate nuclear and cytoplasmic fractions and minimize contamination from the ER^32^,^35^ (Fig. 1A).

Previous studies investigating subcellular localization of RNAi factors had used cells that had not been transfected with siRNAs^32^. By contrast, the T47D and MDA-MB-453 breast cancer cells used in this study had been transfected with duplex RNAs in complex with cationic lipids prior to analysis. To determine whether we obtain sufficiently pure nuclei and cytoplasm from transfected cells, we analyzed cellular fractions after transfecting control siRNA (siCtrl) or siRNAs that were designed to silence AGO1, AGO2, or TRBP (siAGO1, siAGO2, or siTRBP) into T47D cells. We found that Tubulin, a marker for cytoplasm, was almost absent from our nuclear preparations in control- and siRNA-treated samples (Fig. 1B–D). Conversely, Lamin A/C, a marker for nuclei, was almost absent from our cytoplasmic fractions.

RNAi factors can also reside in the ER contiguous with the nuclear membrane^34^, making separation of ER from the nuclear contents a necessity. Analysis of purified nuclei revealed that Calnexin, a marker for ER contamination, was largely absent in control- and siRNA-treated samples. Similar results using Tubulin, Lamin A/C, and Calnexin as markers for purity were obtained for MD-MB-453 cells (Figure S1A). These experiments suggest that our nuclear preparations were largely free of cytoplasmic or ER contamination and that our cytoplasmic preparations are largely free of contamination from nuclei.

### Effect of AGO1 knockdown on distribution of RNAi factors

**Figure 2:**
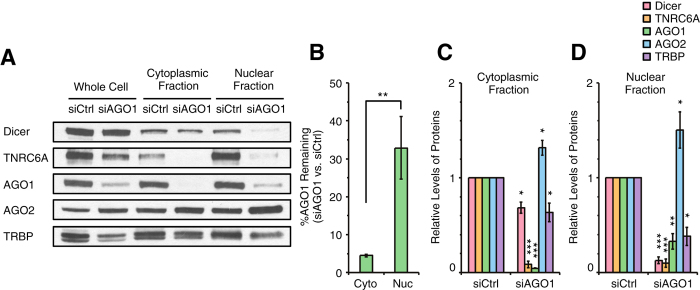
Effect of AGO1 knockdown on levels of other RNAi factors in the cytoplasm and the nucleus. (**A**) Western blot data showing levels of Dicer, TNRC6A, AGO1, AGO2, and TRBP proteins in the whole cell and the cytoplasmic and the nuclear fractions after siCtrl or siAGO1 treatment. siCtrl and siAGO1 were transfected into T47D cells at 25 nM. Equal amount of proteins (25 μg) from each fraction were analyzed by SDS-PAGE. Data were representative from three independent experiments. (**B**) %AGO1 remaining after siAGO1 treatment in the cytoplasm and the nucleus. n = 3. Error bars are SD. **p < 0.01 (unpaired t-test). (**C**,**D**) Quantitation of data shown in panel A and independent replicates (n = 3) showing levels of Dicer, TNRC6A, AGO1, AGO2, and TRBP proteins in the cytoplasmic (**C**) and the nuclear (**D**) fractions after siAGO1 treatment relative to siCtrl treatment. Error bars are SD. *p < 0.05, **p < 0.01, and ***p < 0.001 relative to siCtrl treatment (paired t-test).

**Figure 3:**
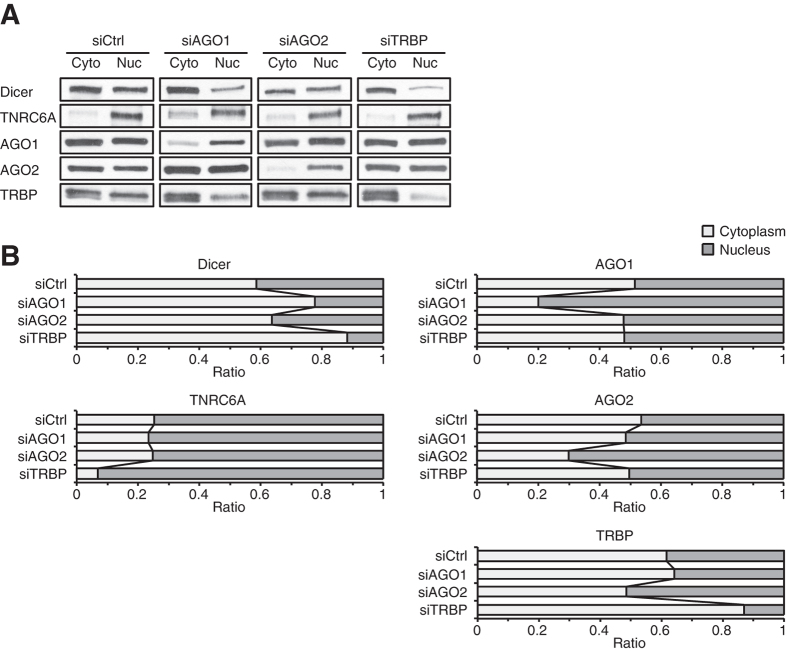
Relative levels of RNAi factors between the cytoplasm and the nucleus in T47D cells. (**A**) Western blot data showing relative levels of Dicer, TNRC6A, AGO1, AGO2, and TRBP between the cytoplasm and the nucleus in siCtrl-, siAGO1, siAGO2, or siTRBP-treated cells. [dsRNA] = 25 nM. The cytoplasmic and nuclear fractions were prepared in the equal amount of buffers. A equal volume of samples from each fraction were analyzed by SDS-PAGE to evaluate relative levels of each protein between the cytoplasm and the nucleus. Data were representative from three independent experiments. (**B**) Ratio of each RNAi factor between the cytoplasm and the nucleus after siCtrl, siAGO1, siAGO2, or siTRBP treatment. Data were averaged from 9 data sets for siCtrl-treated samples and 3 data sets for siAGO1-, siAGO2-, or siTRBP-treated samples.

When siRNAs are used to reduce expression of cellular proteins the efficiency of knockdown is typically evaluated by examining preparations obtained from whole cells. One shortcoming of these analyses is that they do not reveal whether reduced protein levels are more pronounced in one cellular compartment relative to another. In addition, most studies focus on evaluating levels of the RNAi factor targeted by the siRNA and do not examine the possibility that expression of other RNAi factors may also change. To accomplish a broader evaluation of the response of RNAi factors to RNAi-mediated gene knockdown of key components of RISC, we examined how using siRNAs to reduce AGO1 expression affected subcellular distribution of AGO1, AGO2, Dicer, TNRC6A, and TRBP proteins (Figs 2 and 3).

There are four AGO proteins in human cells (AGO1-AGO4). AGO2 possesses catalytic activity and can cleave bound mRNAs^28^,^29^. The function of AGO1 is less well-defined but can affect miRNA-mediated inhibition of translation^36^,^37^, splicing^4^, and transcription^14^. It is possible that AGO1 and AGO2 have overlapping functions or targets where cleavage is not required for regulation.

We find that, in T47D cells transfected with control siRNA, AGO1 is present at similar levels in nuclear and cytoplasmic fractions (Fig. 3). After transfection of anti-AGO1 siRNA into T47D cells, only ~5% of the original level of AGO1 remained in cell cytoplasm. In cell nuclei, however, ~34% remained (Fig. 2B) consistent with a ~7-fold retention of residual AGO1 in the nucleus relative to the cytoplasm. We also observed a similar relative retention of nuclear versus cytoplasmic AGO1 after AGO1 knockdown in MDA-MB-453 cells (Figure S2A).

Silencing AGO1 also affected the levels of the other RNAi factors with TNRC6A being most affected (Fig. 2A,C,D). TNRC6A is an RNA binding protein that associates with AGO^36^. TNRC6A associates with cytoplasmic p-bodies^38^,^39^ and, like AGO, has often been assumed to be localized to cell cytoplasm although it possesses a nuclear localization signal and can be visualized in cell nuclei by microscopy^32^,^40^.

In T47D cells, a majority of TNRC6A protein was observed in the nuclear fraction (Fig. 3). Predominant nuclear localization of TNRC6A is further evidence for the potential of nuclear RNAi to play an important role in controlling cellular processes. When AGO1 expression was silenced, TNRC6A levels were profoundly reduced (~90%) in both nuclei and cytoplasm (Fig. 2A,C,D) indicating a tight regulatory linkage between the two proteins. Silencing AGO1 reduced nuclear Dicer levels by 90% and nuclear TRBP levels by >50%. Dicer and TRBP expression in the cytoplasm was reduced less (40%), reflecting a lower sensitivity to reduction of AGO1 levels (Fig. 2A,C,D).

By contrast to reduced levels of TRBP, Dicer, and TRNC6A, silencing AGO1 led to ~1.4 fold increase in AGO2 in both nuclear and cytoplasmic fractions (Fig. 2A,C,D) A similar increase in AGO2 levels was observed in MDA-MB-453 cells (Figure S2A). Use of a second anti-AGO1, siAGO1-b (Table S1) also yielded similar results (Figure S4). These data raise the possibility that cells may compensate for reduction of AGO1 by increasing expression of AGO2. More broadly, our results show that reduced levels of AGO1 lead to substantial changes in both the expression and intracellular distribution of AGO2 and other RNAi factors. Levels of Dicer, TNRC6A, AGO1, and AGO2 were not substantially changed after transfection with three different negative control dsRNAs (Figure S3).

In the experiments presented in Fig. 2, we analyzed the same amount of total protein from the different fractions by western blot. This allowed us to directly compare levels of RNAi factors between control and siRNA treatments within each fraction. The data, however, do not show the ratio of each protein between the cytoplasm and the nucleus on a “per cell” basis because the amount of total protein in the cytoplasm and nucleus differs.

To evaluate relative amount of each RNAi factor between the cytoplasm and the nucleus per cell, we obtained nuclei and cytoplasm from equal numbers of cells and analyzed equal cell equivalents by western blot (Fig. 3). When AGO1 expression was silenced by anti-AGO1 siRNA, we confirmed that AGO1 was preferentially retained in cell nuclei. This analysis also confirmed that silencing of AGO1 led to reduced levels of nuclear Dicer.

### AGO2 is preferentially retained in the nucleus after AGO2 knockdown

**Figure 4:**
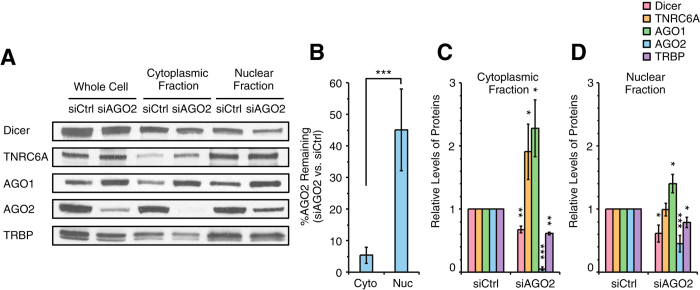
Effect of AGO2 knockdown on levels of other RNAi factors in the cytoplasm and the nucleus. (**A**) Western blot data showing levels of Dicer, TNRC6A, AGO1, AGO2, and TRBP proteins in the whole cell and the cytoplasmic and the nuclear fractions after siCtrl or siAGO2 treatment. siCtrl and siAGO2 were transfected into T47D cells at 25 nM. Equal amount of proteins (25 μg) from each fraction were analyzed by SDS-PAGE. Data were representative from three independent experiments. (**B**) %AGO2 remaining after siAGO2 treatment in the cytoplasm and the nucleus. n = 3. Error bars are SD. ***p < 0.001 (unpaired t-test). (**C, D**) Quantitation of data shown in panel A and independent replicates (n = 3) showing levels of Dicer, TNRC6A, AGO1, AGO2, and TRBP proteins in the cytoplasmic (**C**) and the nuclear (**D**) fractions after siAGO2 treatment relative to siCtrl treatment. Error bars are SD. *p < 0.05, **p < 0.01, and ***p < 0.001 relative to siCtrl treatment (paired t-test).

AGO2 is the catalytic engine for cytoplasmic RNAi and the primary AGO responsible for most experimental gene silencing. Because of the known importance of AGO2 in cytoplasmic RNAi we also examined how siRNA-mediated reduction of AGO2 expression would affect distribution of Dicer, TNRC6A, AGO1, AGO2, and TRBP in the nucleus and the cytoplasm (Fig. 4A).

We observed that AGO2 is present in slightly lower amounts in nuclear extracts relative to cytoplasmic extracts after treatment with control siRNA (Fig. 3). After transfection of anti-AGO2 siRNA and knockdown of AGO2 protein levels, only ~5% of AGO2 protein remained in the cytoplasm (Fig. 4B). In nuclear extracts, however, amounts of AGO2 after treatment of anti-AGO2 siRNA remained at ~45% of pre-treatment levels. This residual protein represents a 9-fold retention of nuclear AGO2. We observed a similar relative retention of AGO2 in nuclear extract from MDA-MB-453 cells (Figure S2B). Analysis of AGO2 levels on a per cell basis confirmed retention of AGO2 in cell nuclei relative to cytoplasm after silencing AGO2 expression with siRNAs (Fig. 3).

We examined how reduced levels of AGO2 affected distribution of RNAi factors Dicer, TNRC6A, AGO1, AGO2, and TRBP (Fig. 4). We observe only moderately reduced nuclear Dicer expression (~40%), a sharp contrast to the 90% reduction of nuclear Dicer upon silencing AGO1 expression. Another marked difference in outcomes between silencing AGO1 and AGO2 was our observation that TNRC6A levels in both nuclei and cytoplasm are stable or increased after reduction of AGO2 expression (Fig. 4A,C,D). This finding is consistent with the conclusion that expression of TNRC6A is linked differently with AGO1 versus AGO2.

After reducing AGO1 expression we observed an increase in AGO2 protein levels (Fig. 2 and S2A). We now observed that reducing AGO2 expression led to an ~1.4–2.3-fold increase in AGO1 (Fig. 4A,C,D). A similar increase of AGO1 expression upon siAGO2 treatment was observed in MDA-MB-453 cells (Figure S2B). These observations further suggest a potential for AGO1 and AGO2 expression to be balanced in a matter that allows one AGO to at least partially compensate for reduction of the other.

### Microscopy confirms nuclear retention of AGO2

**Figure 5:**
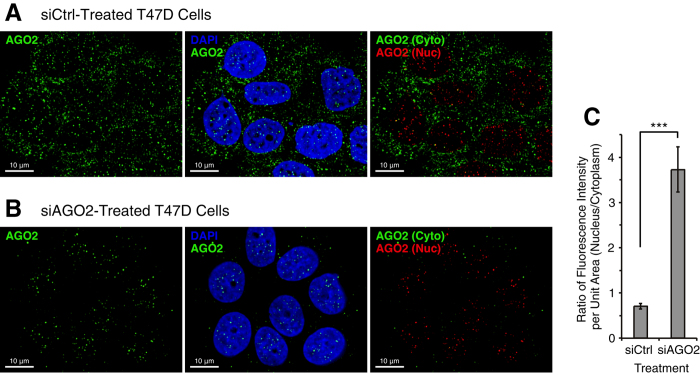
Immunofluorescence microscopy reveals nuclear retention of AGO2 after AGO2 depletion by siRNA. (**A**) Immunofluorescence microscopic images for AGO2 in T47D cells after siCtrl transfection. [siCtrl] = 25 nM. (**B**) Immunofluorescence microscopic images for AGO2 in T47D cells after siAGO2 transfection. [siAGO2] = 25 nM. The images were taken 3 days after transfection of duplex RNAs. Z-sections in the middle of the cells (10 image slices, interval: 0.2 μm) were stacked and projected three-dimensionally. left: AGO2 (green); middle: DAPI (blue) and AGO2 (green); right: cytoplasmic AGO2 (green) and nuclear AGO2 (red). Using Imaris program, fluorescence signals which overlap DAPI’s blue signals are shown in red. The scale bar = 10 μm. See also Figure S6 for additional images. (**C**) Ratio of fluorescence intensity per unit area in the nucleus relative to the cytoplasm. n = 16. Error bars are SEM. ***p < 0.001 (unpaired t-test).

To further investigate the nuclear retention of AGO2 inside cells we used wide-field immunofluorescence microscopy. After cells were treated with anti-AGO2 siRNA or a noncomplementary control RNA (siCtrl), cells were fixed and then immunostained using anti-AGO2 antibody to examine subcellular distribution of AGO2. Microscopic observation of siCtrl-treated cells revealed AGO2 distributed throughout both cytoplasm and nucleus (Fig. 5A and S6A), consistent with the previous report for T47D cells that had not been transfected with any siRNA^32^.

After siRNA-mediated knockdown of AGO2, fluorescence showed a shift in favor of signal from cell nuclei (Fig. 5B,C and S6B). Taken together, data from western analysis and immunofluorescence microscopy indicate that the residual AGO2 is preferentially retained in cell nuclei after overall cellular levels of AGO2 are reduced. Commercially available anti-AGO1 antibodies (Cell Signaling Technology #5053, Wako #015-22411) did not possess sufficient affinity or specificity to obtain similar definitive immunofluorescence microscopic images for AGO1 localization.

### Effect of reducing levels of TRBP

**Figure 6:**
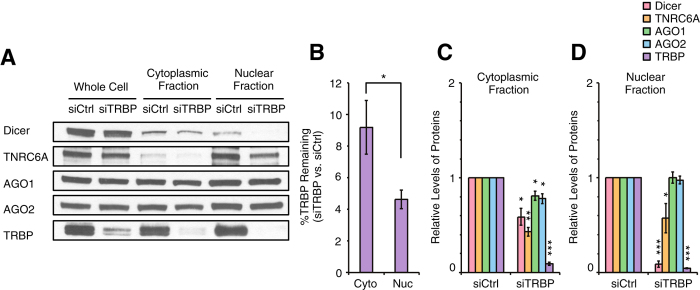
Effect of TRBP knockdown on levels of other RNAi factors in the cytoplasm and the nucleus. (**A**) Western blot data showing levels of Dicer, TNRC6A, AGO1, AGO2, and TRBP proteins in the whole cell and the cytoplasmic and the nuclear fractions after siCtrl or siTRBP treatment. siCtrl and siTRBP were transfected into T47D cells at 25 nM. Equal amount of proteins (25 μg) from each fraction were analyzed by SDS-PAGE. Data were representative from three independent experiments. (**B**) %TRBP remaining after siTRBP treatment in the cytoplasm and the nucleus. n = 3. Error bars are SD. *p < 0.05 (unpaired t-test). (**C,D**) Quantitation of data shown in panel A and independent replicates (n = 3) showing levels of Dicer, TNRC6A, AGO1, AGO2, and TRBP proteins in the cytoplasmic (**C**) and the nuclear (**D**) fractions after siTRBP treatment relative to siCtrl treatment. Error bars are SD. *p < 0.05, **p < 0.01, and ***p < 0.001 relative to siCtrl treatment (paired t-test).

We also examined how siRNA-mediated reduction of TRBP expression would affect distribution of Dicer, TNRC6A, AGO1, AGO2, and TRBP in the nucleus and cytoplasm (Fig. 6 and S5). TRBP is a double-stranded RNA binding protein that associates with other RNAi factors to form a minimal RNAi induced silencing complex (RISC)^41^,^42^. TRBP exists in mammalian cell nuclei^32^, and may play a role in regulating transcription^43^.

We observed that TRBP was present at similar levels in nuclear and cytoplasmic extracts in T47D cells (Fig. 3). Unlike silencing AGO1 or AGO2, which led to relative retention of AGO proteins in the nucleus, we observed that treatment with anti-TRBP siRNA reduced TRBP levels modestly more efficiently in the nucleus relative to cytoplasm (~9% of original cytoplasmic levels and 5% of nuclear levels) (Fig. 6B).

The most striking change in distribution of other RNAi factors was observed for Dicer, the enzyme responsible for processing pre-miRNAs^44^. In cells treated with control siRNA, Dicer was evenly distributed between cytoplasm and nuclear fractions (Fig. 3). When T47D cells were treated with anti-TRBP siRNA, levels of cytoplasmic Dicer were reduced by 40% (Fig. 6A,C). In nuclear extracts, however, less than 5% of original levels of Dicer protein were detected (Fig. 6A,D) revealing a more substantial impact. TNRC6A levels were modestly reduced in both cytoplasmic and nuclear fractions. AGO1 and AGO2 levels were slightly decreased in the cytoplasm and unchanged in nuclear fractions (Fig. 6A,C,D). These data suggest that, with exception of Dicer, levels and subcellular distribution of RNAi factors are less sensitive to reduction of TRBP than they were to reduction of AGO1 or AGO2.

### Immunofluorescence microscopy of TRBP

**Figure 7:**
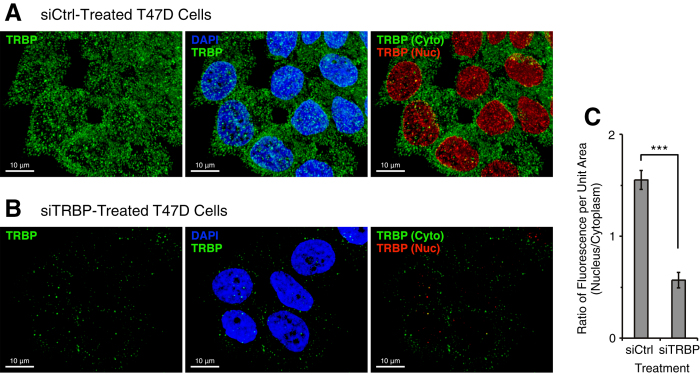
Immunofluorescence microscopy for TRBP after control siRNA or siTRBP treatment. (**A**) Immunofluorescence microscopic images for TRBP in T47D cells after siCtrl transfection. [siCtrl] = 25 nM. (**B**) Immunofluorescence microscopic images for TRBP in T47D cells after siTRBP transfection. [siTRBP] = 25 nM. The images were taken 3 days after transfection of duplex RNAs. Z-sections in the middle of the cells (10 image slices, interval: 0.2 μm) were stacked and projected three-dimensionally. left: TRBP (green); middle: DAPI (blue) and TRBP (green); right: cytoplasmic TRBP (green) and nuclear TRBP (red). Using Imaris program, fluorescence signals which overlap DAPI’s blue signals are shown in red. The scale bar = 10 μm. See also Figure S6 for additional images. (**C**) Ratio of fluorescence intensity per unit area in the nucleus relative to the cytoplasm. n = 25. Error bars are SEM. ***p < 0.001 (unpaired t-test).

We performed immunofluorescence microscopy as an independent method to evaluate the effect of silencing TRBP on nuclear and cytoplasmic localization of residual protein. When T47D cells were treated with control siRNA we observed distribution of TRBP in the cytoplasm and nucleus, consistent with our western blot analysis (Figs 7A and S6C). We then examined localization of residual TRBP after transfection of an siRNA targeting TRBP. In contrast to the relative enhancement of nuclear AGO2 observed after knockdown of AGO2 (Fig. 5) we observed a modest decrease in the relative levels of nuclear versus cytoplasmic TRBP (Figs 7B,C and S6D). These data show that enhanced nuclear localization is not a universal response to knockdown of RNAi factors.

## Discussion

RNAi has the potential to drive the regulation and recognition of cellular RNAs, not only in the cytoplasm of mammalian cells but also in the nucleus^24^. The extent of gene regulation by small RNAs and RNAi factors in cell nuclei, however, has remained obscure. We report here that, not only do RNAi factors exist in cell nuclei, but when key RNAi factors AGO1 or AGO2 are depleted individually, the remaining AGO1 or AGO2 is preferentially retained in nuclei and levels of other RNAi factors in the cytoplasm and/or nucleus were altered. Our data of AGO depletion and subcellular distribution have implications for both experimental science and basic biology.

For experimental science, one approach to understanding endogenous roles for AGO1 or AGO2 within cells is to use siRNAs to deplete protein and then examine the consequences. Our data indicated investigations of the nuclear role of AGO1 or AGO2 may not always be straightforward because under depleted conditions cells will tend to retain remaining AGO in their nuclei. When levels of knockdown are measured by examining the whole cell, false assumptions about the levels of nuclear AGO may lead to inaccurate conclusions. For example, addition of an anti-AGO siRNA may produce a profound reversal in the cytoplasm while being substantially less effective in the nucleus. If maximal knockdown of nuclear AGO is required, it is possible that creation of genomic AGO knockout cells might be a necessary choice for some experiments where the more convenient siRNA-mediated knockdown is not adequate.

Further complicating matters, we find that reduction of AGO1 or AGO2 changes expression of other RNAi factors. Most directly, reduction of AGO1 or AGO2 expression increases expression of the other AGO variant. For some biological processes, this increased expression may at least partially compensate for reduced expression of the targeted AGO and lead to a less robust physiologic outcome. It is possible, therefore that for some applications knockout or knockdown of both AGO1 and AGO2 might be necessary to reveal the full impact of loss of AGO activity. Cells also express two other AGO variants, AGO3 and AGO4. These variants have been much less well studied than AGO1 or AGO2. AGO3 and AGO4 are expressed at much lower levels than AGO1 and AGO2^16^. These low expression levels suggest that their impact on cell physiology will be less, but more investigation will be needed of these more rare AGO variants. Finally, knockdown of AGO1 has a major impact on levels of nuclear Dicer, TNRC6A, and TRBP in the cytoplasm and nucleus relative to knockdown of AGO2. Changes in the expression of these proteins may also affect interpretation of some gene silencing experiments, especially those specifically relating to AGO1 (and, to a lesser extent AGO2).

For basic biology, our findings reveal connections between expression of key RNAi components. Our finding that knockdown of AGO1 enhances AGO2 expression, and vice versa, suggests that cells may have mechanisms to compensate for changes related to the two most highly expressed AGO variants. The substantial changes in Dicer, TNRC6A, and TRBP expression upon reduction of AGO1 expression may be because AGO1 stimulates their expression either directly or indirectly through miRNA pathways. Alternatively, AGO1 may play a role in transporting the other RNAi factors into the nucleus or maintaining their stability through protein-protein interactions.

One interesting point from the knockdown experiments is that AGO1 and AGO2 knockdown made an opposite impact on TNRC6A protein levels. AGO1 knockdown reduced TNRC6A levels by more than 90% in both the cytoplasm and the nucleus, while AGO2 knockdown increased TNRC6A levels especially in the cytoplasm (Figs 2 and 4). This finding may implicate different modes of protein interactions between AGO1 and AGO2 inside cells.

The Medipal domain within TRBP binds to the ATPase/helicase domain within Dicer^45^,^46^ and the interaction enhances Dicer-catalyzed miRNA-processing kinetics^47^. Different research groups previously performed TRBP knockdown experiments and discussed the effect of TRBP depletion on Dicer stability and miRNA-processing^45^,^48^. They analyzed Dicer levels in either FLAG-AGO2 immunoprecipitates or total cell extracts from HeLa cells upon TRBP knockdown and the data were not perfectly consistent. Our experiments using subcellular fractions from T47D cells showed that, like AGO1 depletion greatly reduced nuclear Dicer, TRBP depletion by anti-TRBP siRNA also reduced Dicer levels especially in the nucleus (Fig. 6). It appears that nuclear Dicer levels are tightly coupled by cellular levels of AGO1 and TRBP. Interestingly, a previous study has shown that the relative subcellular distribution of Dicer in cytoplasm versus nucleus can vary between cell lines^23^.

Transcription and splicing are critical biological processes that occur in cell nuclei and involve RNA. Using designed RNAs targeting specific sequences it is clear that nuclear RNAi machinery can direct transcriptional silencing, transcriptional activation, and alternative splicing. Our data, however, suggest that the landscape of expression and distribution of nuclear RNAi factors is unexpectedly complex.

## Methods

### Cell culture and siRNA transfection

T47D cells (ATCC) were cultured in RPMI-1640 supplemented with 10% FBS, 0.5% MEM non-essential amino acids (Sigma), 1 mM HEPES (Sigma), 1 mM sodium pyruvate (Sigma), and 200 U/mL human insulin at 37 °C in 5% CO_2_. MDA-MB-453 cells (ATCC) were cultured in Dulbecco’s Modified Eagle’s Medium supplemented with 10% FBS. Reverse transfection was performed to deliver siRNAs into cells (3 million cells/15 cm dish for T47D and 5 million cells/15 cm dish for MDA-MB-453; two dishes for each treatment) using Lipofectamine RNAiMAX. Three days later, cells were harvested for subcellular fractionation. Sequences of the siRNAs used in this study are listed in Table S1.

### Subcellular fractionation

For subcellular fractionation, the protocol reported previously^32^,^35^ was modified and used in this study. siRNA-treated cells were harvested by scraping from dishes on Day 3. A portion of the cells were lysed with cell lysis buffer (50 mM Tris-HCl, 120 mM NaCl, 0.5% NP-40, 1 mM EDTA, 1 mM DTT, and 1X protease inhibitor (Calbiochem)). The samples were frozen and thawed repeatedly and then centrifuged at 13,000 rpm at 4 °C for 10 min. The supernatants were kept as whole cell samples. The rest of the cells from the dishes were lysed with 0.8 ml of hypotonic lysis buffer (10 mM Tris-HCl (pH 7.5), 10 mM NaCl, 3 mM MgCl_2_, 0.5% NP-40, 1X protease inhibitor) and centrifuged at 500Xg at 4 °C for 10 min. This supernatant was kept as cytoplasmic extract. Nuclear pellets were washed with 0.4 ml of hypotonic lysis buffer again and the supernatant was combined (total cytoplasmic extracts: 1.2 ml). Nuclear pellets were washed with hypotonic lysis buffer one more time to minimize ER contamination. To prepare nuclear extracts, nuclear pellets were resuspended in 1.2 ml of nuclear lysis buffer (50 mM Tris-HCl (pH 8.1), 10 mM EDTA, 1% SDS, 1X protease inhibitor) and then sonicated (20% power, 20 sec, 2 pulses). The samples were centrifuged at 13,000 rpm at 4 °C for 10 min and the supernatant was kept as nuclear extracts (1.2 ml).

### Western blot analysis

Protein concentrations in whole cell, cytoplasmic, and nuclear samples were determined using the BCA assay kit (Thermo Scientific). For evaluating relative levels of specific proteins between different treatments in each fraction, 25 μg of total protein from each fraction were analyzed by SDS-PAGE (4–20% TGX gels (Bio-Rad)) (Figs 1, 2, 4, 6, S1, S2, S4B, and S4C). For evaluating ratios of protein between the cytoplasm and the nucleus per cell, an equal amount (volume) of samples taken from 1.2 ml of cytoplasmic or nuclear extracts was also analyzed by western blot (Fig. 3). Gels were run at 110 V for 75 min. After gel electrophoresis, proteins were transferred to nitrocellulose membrane (Amersham Protran; GE Healthcare) at 100 V for 1 h 30 min. After blocking the membrane with 5% non-fat dry milk/TBST at room temperature for 1 h, the membrane was incubated with primary antibodies at the following dilution ratio: anti-Dicer antibody (abcam; ab14601; 1:1000), anti-TNRC6A(GW182) antibody (Bethyl Labs; A302-329A; 1:8000), anti-AGO1 antibody (Cell Signaling; #5053; 1:2000), anti-AGO2 antibody (Wako; 015-22411; 1:1500), anti-TRBP antibody (from Dr. Qinghua Liu’s Lab; 1:500), anti-Tubulin antibody (Sigma; T5201; 1:5000), anti-Lamin A/C antibody (abcam; ab8984; 1:2000), and anti-Calnexin antibody (Cell Signaling; #2433; 1:5000). HRP-conjugated anti-mouse IgG or rabbit IgG (1:1000–10000; Jackson ImmunoResearch) secondary antibody was used for visualizing proteins using SuperSignal West Pico Chemiluminescent Substrate (Thermo Scientific). Protein bands were quantified using ImageJ software. Original western blot images are presented in Figure S7.

### Immunofluorescence microscopy

T47D cells were reverse-transfected with siCtrl, siAGO2, or siTRBP at 25 nM on 35 mm dishes with a 14 mm glass bottom. Three days after transfection, cells were fixed with 4% paraformaldehyde for 15 min, washed with PBS three times, and then permeabilized with 70% ethanol. Cells were incubated with anti-AGO2 antibody (Wako; 1:100) in PBS/1% normal goat serum (NGS) or anti-TRBP antibody (from Dr. Qinghua Liu; 1:100) in PBS/5% NGS overnight. Cells were washed with PBS three times and then incubated with Alexa Fluor 488 goat anti-mouse IgG antibody in PBS/1% NGS (for AGO2) or Alexa Fluor 594 goat anti-rabbit IgG antibody in PBS/5% NGS (for TRBP) for 1 h. Cells were washed with PBS three times and then set in mounting medium with DAPI. Cells were imaged by wide-field epifluorescence microscopy and images processed by blind deconvolution with AutoQuant X3 (Media Cybernetics). Using Imaris (Bitplane), Z-sections from the middle of the cells (10 image slices; interval: 0.2 μm) were stacked and 3-dementionally projected. Fluorescence signals from AGO2 or TRBP which overlap DAPI’s blue signals were taken as nuclear AGO2 or TRBP and the green color was changed into red, while the other signals were taken as cytoplasmic AGO2 or TRBP (Figs 5A,B, 7A,B and S6).

## Additional Information

**How to cite this article**: Matsui, M. *et al.* Reduced Expression of Argonaute 1, Argonaute 2, and TRBP Changes Levels and Intracellular Distribution of RNAi Factors. *Sci. Rep.* **5**, 12855; doi: 10.1038/srep12855 (2015).

## Supplementary Material

Supplementary Information
