# Supplementary material for: Reduced Expression of Argonaute 1, Argonaute 2, and TRBP Changes Levels and Intracellular Distribution of RNAi Factors
Source: Sci Rep. 2015 Aug 5;5:12855. doi: 10.1038/srep12855 (PMC4525381; doi:10.1038/srep12855)
Supplement: Supplementary Information [file srep12855-s1.pdf]

Supplementary Information

Reduced Expression of Argonaute 1, Argonaute 2, and TRBP Changes Levels and Intracellular Distribution of RNAi Factors

Masayuki Matsui, Liande Li, Bethany A. Janowski, and David R. Corey*
Departments of Pharmacology and Biochemistry, University of Texas Southwestern Medical Center, Dallas, Texas, 75390-9041.

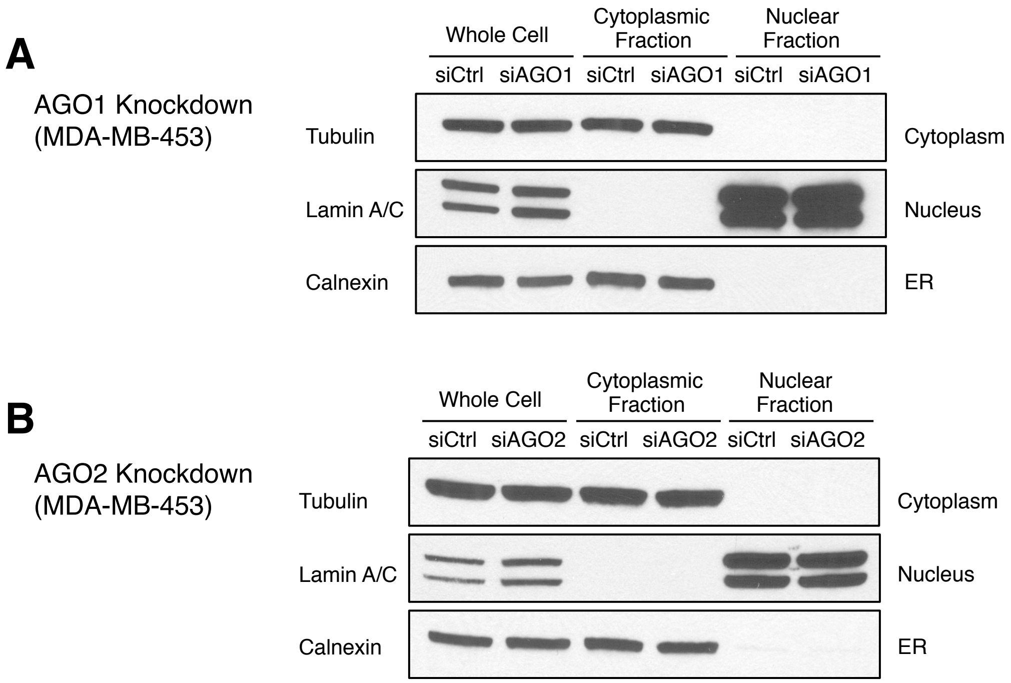

Figure S1, Related to Figure 1. Subcellular fractionation of MDA-MB-453 to isolate whole cells, cytoplasmic fractions, and nuclear fractions. (**A, B**) Western blot analysis for Tubulin (cytoplasmic marker), Lamin A/C (nuclear marker), and Calnexin (ER marker) showing the purity of each fraction prepared from siCtrl-, siAGO1- or siAGO2-treated MDA-MB-453 cells. siRNAs specific for AGO1 or AGO2 were transfected into cells at 25 nM using Lipofectamine RNAiMAX. Cells were harvested 3 days after transfection followed by subcellular fractionation. Equal amount of proteins (25 μg) from each fraction was analyzed by SDS-PAGE (4–20% TGX gels).

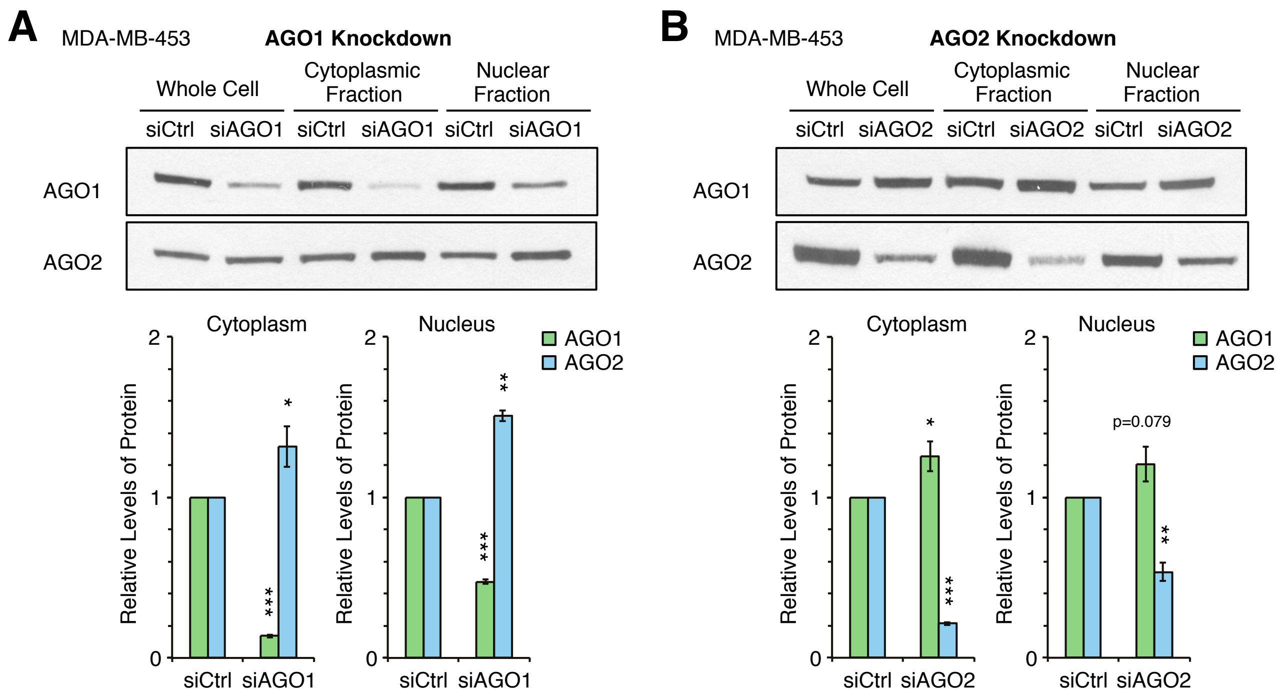

Figure S2, Related to Figures 2 and 4. Nuclear retention of AGO1/AGO2 after siRNA treatments and compensatory effects of AGO1/AGO2 expression in MDA-MB-453 cells. (**A, B**) Western blot data for AGO1 and AGO2 in the whole cell and the cytoplasmic and the nuclear fractions after siAGO1 or siAGO2 treatment (upper) and the quantitation of AGO1 or AGO2 protein levels (lower, n=3). siCtrl, siAGO1, and siAGO2 were transfected into MDA-MB-453 cells at 25 nM. Equal amount of proteins (25 μg) from each fraction were analyzed by SDS-PAGE. Error bars are SD. *p<0.05, **p<0.01, and ***p<0.001 relative to siCtrl treatment (paired t-test).

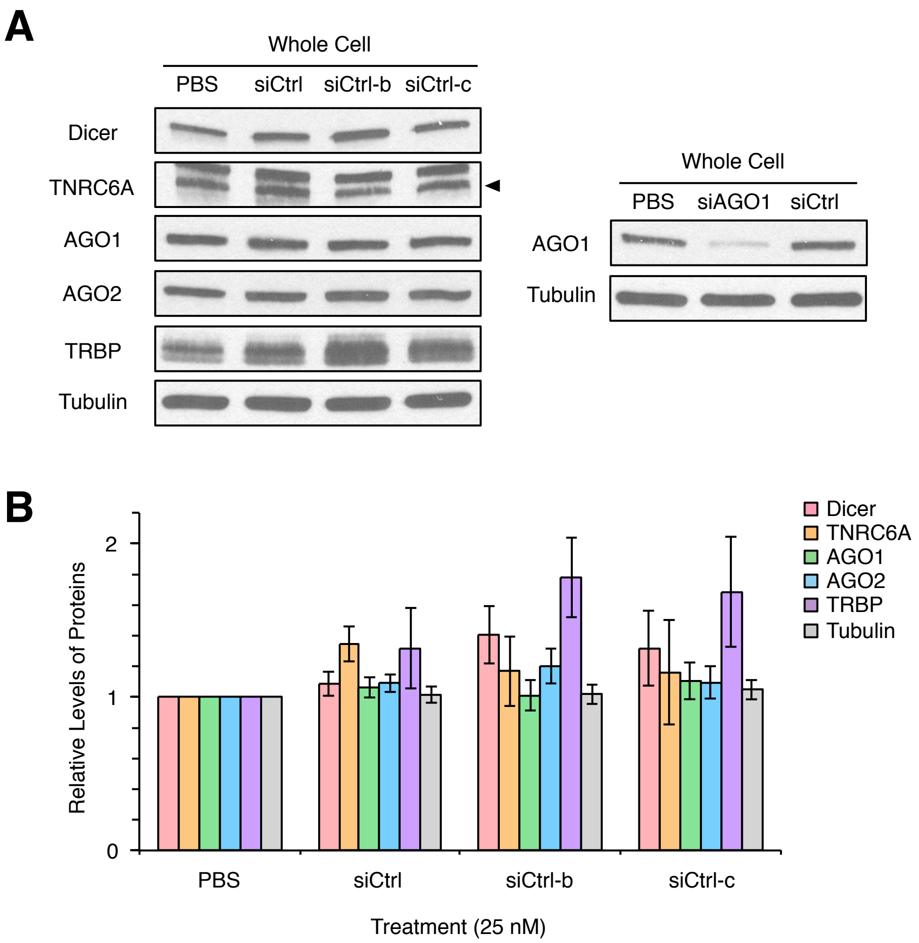

Figure S3. Effect of negative control dsRNAs on expression of RNAi factors. (**A**) Western blot data showing levels of Dicer, TNRC6A, AGO1, AGO2, TRBP, Tubulin proteins in the whole cell samples treated with 3 different non-complementary negative dsRNA controls (siCtrl, siCtrl-b, siCtrl-c) (left). siAGO1 was used as positive control to check transfection efficiency (right). dsRNAs were transfected into T47D cells at 25 nM using Lipofectamine RNAiMAX. Cells were harvested 3 days after transfection. Whole cell samples (20 µg) were analyzed by SDS-PAGE (4–20% TGX gels). (**B**) Quantitation of western blot data from 3–6 independent experiments.

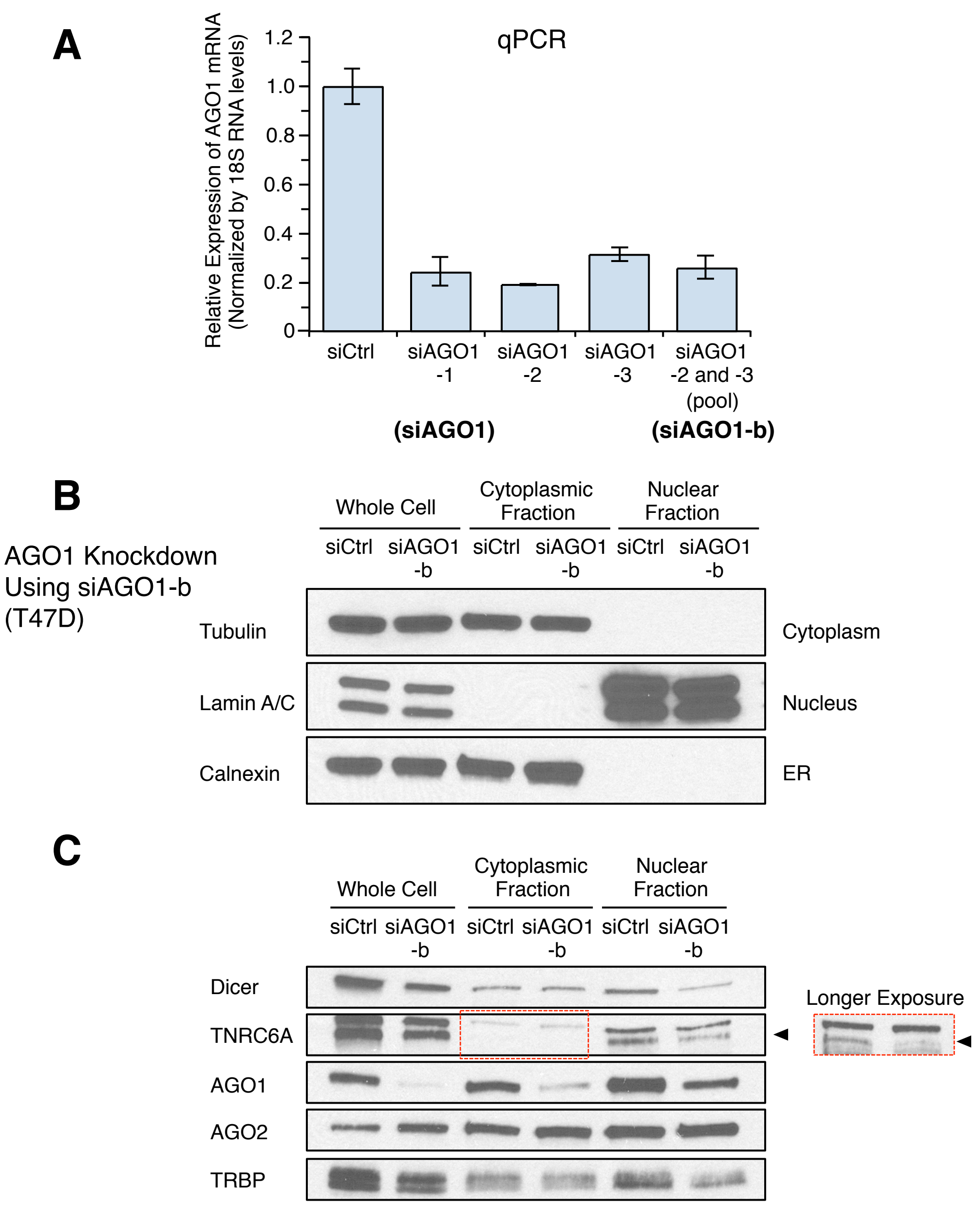

Figure S4, Related to Figure 2. Knockdown of AGO1 using another set of siAGO1 (siAGO1-b) in T47D cells, subcellular fractionation, and western blot analysis of RNAi factors. (**A**) qPCR analysis showing potency of three different siAGO1(-1, -2, -3) and a pool (-2/-3) (25 nM) for AGO1 knockdown. Two days after transfection, cells were harvested using TRIzol and total RNAs were isolated. RT reaction was performed using High Capacity cDNA Reverse Transcription Kit. qPCR was performed using iTaq supermix or iTaq SYBR supermix (Biorad) and primers specific for AGO1 mRNA (Hs00201864_m1) or 18S RNA (Forward: 5'-GACCAGAGCGAAAGCATTTG-3', Reverse: 5'-TCGGAACTACGACGGTATCT-3'). n=2. (**B**) Western blot analysis for Tubulin (cytoplasmic marker), Lamin A/C (nuclear marker), and Calnexin (ER marker) showing the purity of each fraction prepared from siCtrl- or siAGO1-b-treated T47D cells. (**C**) Western blot data showing levels of Dicer, TNRC6A, AGO1, AGO2, and TRBP proteins in the whole cell and the cytoplasmic and the nuclear fractions after siCtrl or siAGO1-b treatment. siCtrl and siAGO1-b were transfected into cells at 25 nM using Lipofectamine RNAiMAX. Cells were harvested 3 days after transfection followed by subcellular fractionation. Equal amount of proteins (25 µg) from each fraction was analyzed by SDS-PAGE (4–20% TGX gels). Data were representative from two independent experiments.

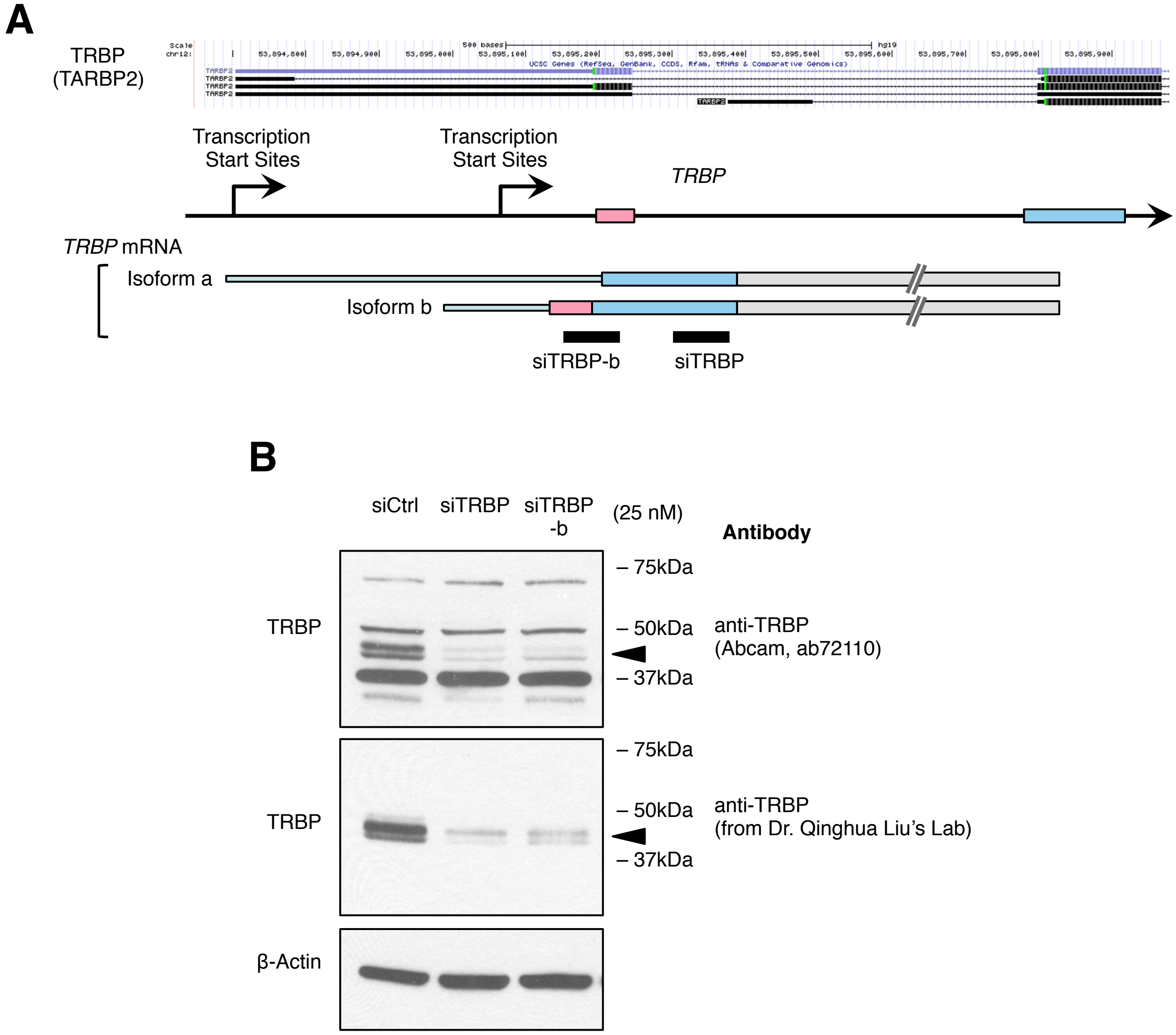

Figure S5, Related to Figures 1 and 6. Potency of two different siTRBPs and specificity of two different anti-TRBP antibodies. (**A**) Scheme of TRBP (TARBP2) gene. Several different transcript isoforms are produced from different promoters on the gene and. One siRNA (siTRBP) was designed to target both TRBP mRNA isoform a (NM_134323.1) and b (NM_134324.2). Another siRNA (siTRBP-b) was designed to specifically target TRBP mRNA isoform b by targeting the exon1/2 junction of isoform b. (**B**) Western blot data showing reduction of TRBP protein after siTRBP or siTRBP-b transfection at 25 nM into T47D cells. Two rabbit polyclonal anti-TRBP antibodies from Abcam (ab72110, upper) and Dr. Qinghua Liu's Lab (middle) were tested to evaluate knockdown efficiency of each siRNA and specificity of these antibodies.

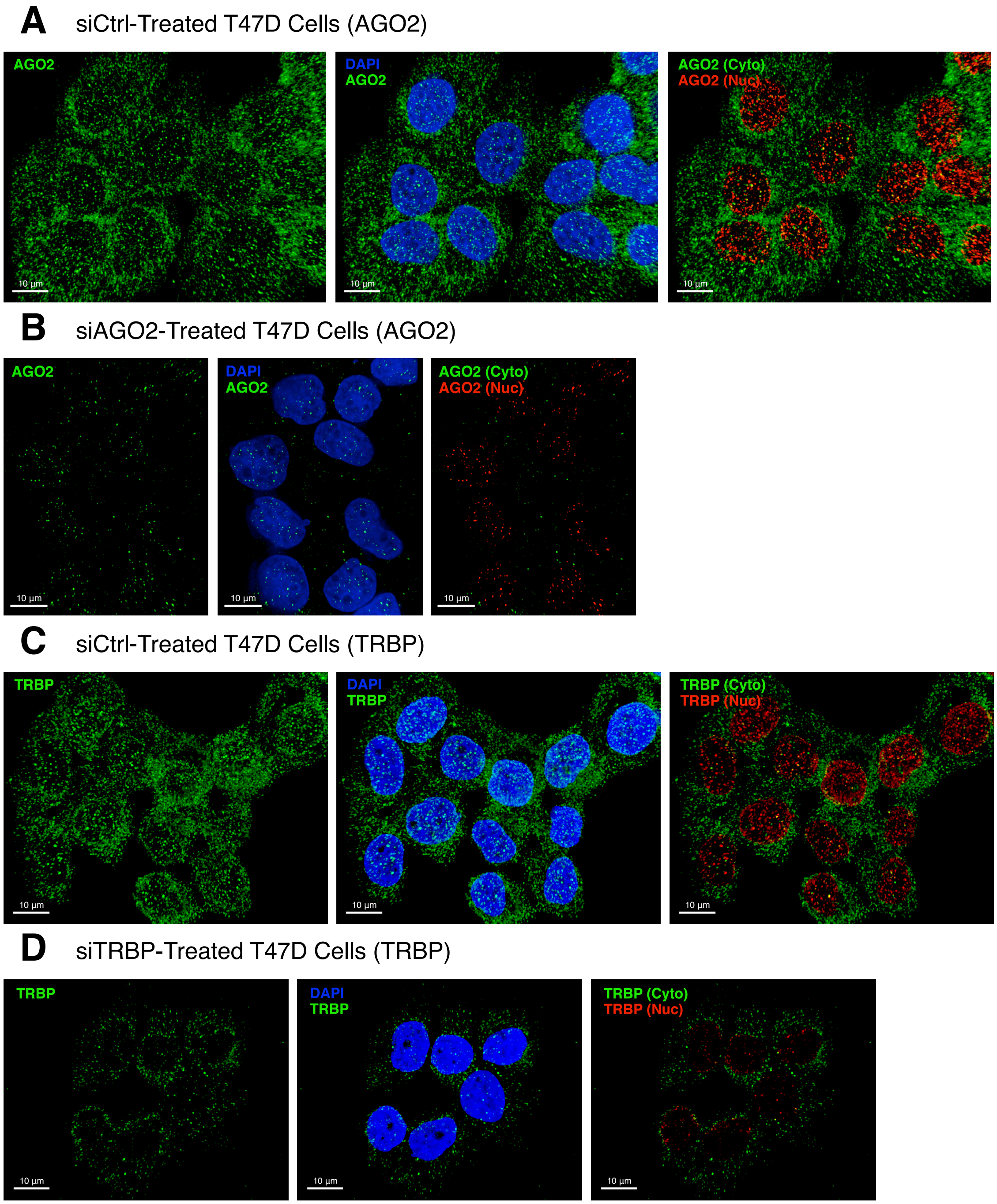

Figure S6, Related to Figures 5 and 7. Immunofluorescence microscopic images for AGO2 and TRBP in T47D cells. (**A, B**) Immunofluorescence microscopic images for AGO2 in T47D cells after siCtrl (**A**) and siAGO2 (**B**) transfection. left: AGO2 (green); middle: DAPI (blue) and AGO2 (green); right: cytoplasmic AGO2 (green) and nuclear AGO2 (red). (**C**,**D**) Immunofluorescence microscopic images for TRBP in T47D cells after siCtrl (**C**) and siTRBP (**D**) transfection. left: TRBP (green); middle: DAPI (blue) and TRBP (green), right: cytoplasmic TRBP (green) and nuclear TRBP (red). Using Imaris program, fluorescence signals which overlap DAPI's blue signals are shown as red. [siRNA]=25 nM. The images were taken 3 days after transfection of duplex RNAs. Z-sections in the middle of the cells (10 image slices, interval: 0.2 µm) were stacked and projected three-dimentionally. The scale bar = 10 µm.

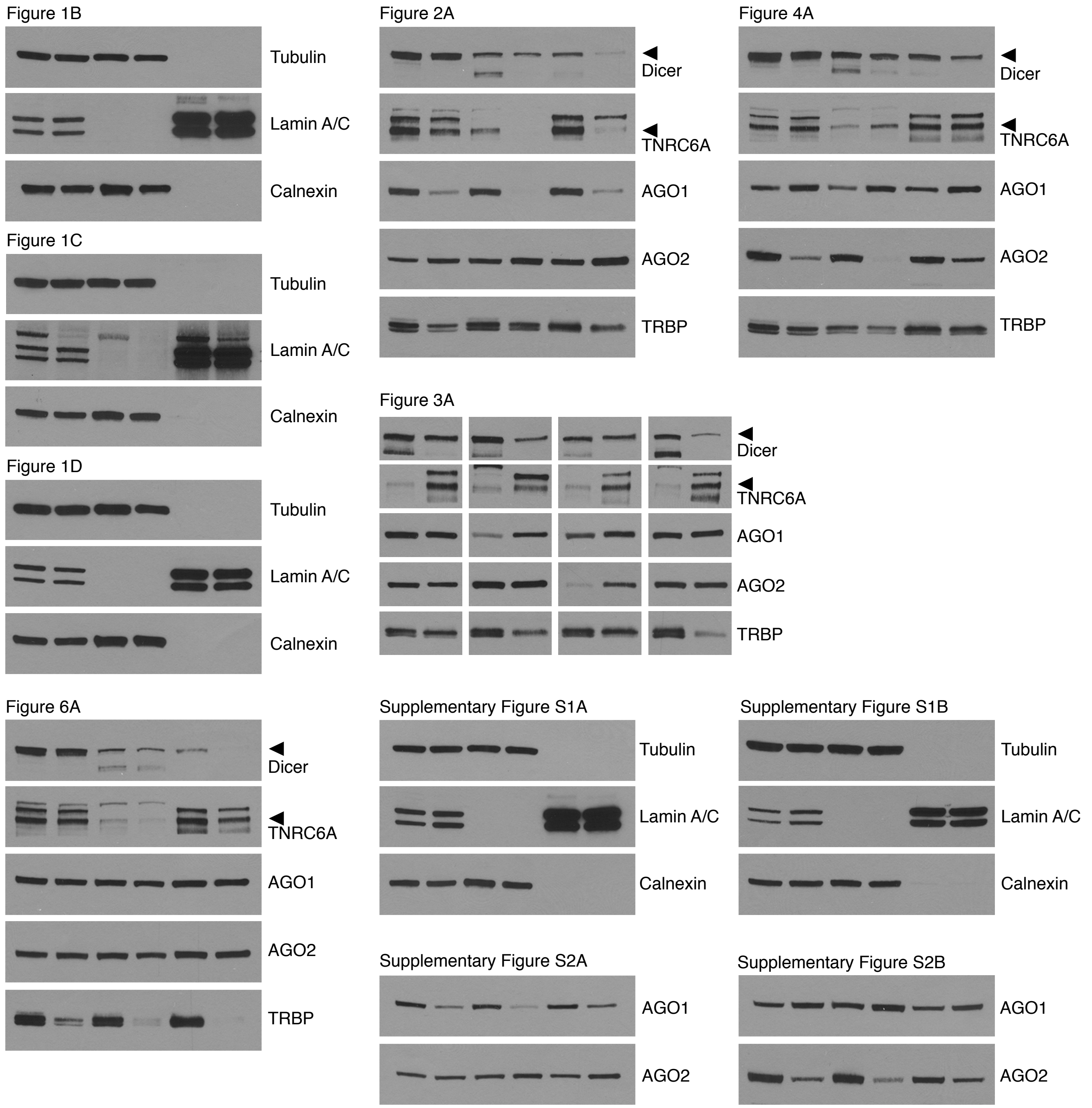

Figure S7, Related to Figures 1, 2, 3, 4, 6, S1, S2, S3, S4, and S5. Original image data for western blot.

Supplementary Figure S3A

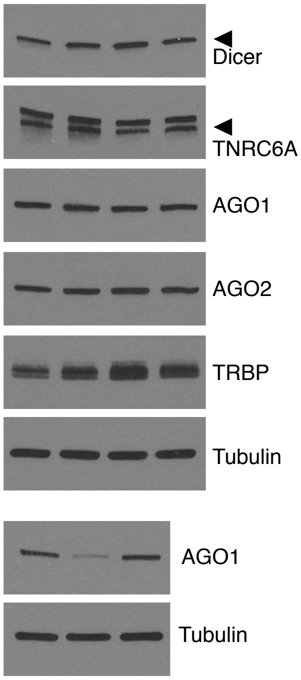

Supplementary Figure S4B

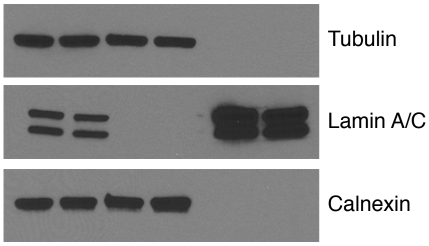

Supplementary Figure S4C

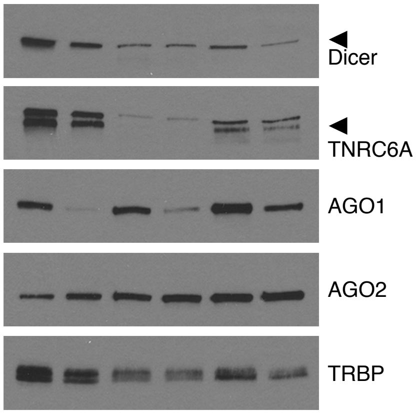

Supplementary Figure S5B

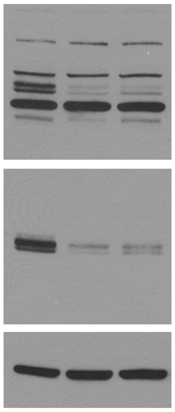

Figure S7 (Continued)

Table S1. Sequences of duplex RNAs used in this research.

Strand Name	dsRNA Name	Sequence (5' → 3')	Target
siCtrl-1 (sense)	siCtrl	UCGAAGUAUUCCGCGUACGdTdT	(Negative Control)
siCtrl-1 (antisense)		CGUACGCGGAAUACUUCGAdTdT	
siCtrl-2 (sense)	siCtrl-b	UCGUCAGUGGAGUCAGAGUdTdT	(Negative Control)
siCtrl-2 (antisense)		ACUCUGACUCCACUGACGAdTdT	
siCtrl-3 (sense)	siCtrl-c	ACUACUGAGUGACAGUAGAUU	(Negative Control)
siCtrl-3 (antisense)		UCUACUGUCACUCAGUAGUUU	
siAGO1-1 (sense)	siAGO1	GGAGUUACUUUCAUAGCAUUU	AGO1 mRNA
siAGO1-1 (antisense)		pAUGCUAUGAAAGUAACUCCUU	
siAGO1-2 (sense)	siAGO1-b (pool)	CAUACACACAGGUGUCUCAUU	AGO1 mRNA
siAGO1-2 (antisense)		pUGAGACACCUGUGUGUAUGUU	
siAGO1-3 (sense)		ACUUAAGGCACUAUGGCACUU	
siAGO1-3 (antisense)		pGUGCCAUAGUGCCUUAAGUUU	
siAGO2-1 (sense)	siAGO2 (pool)	GCACGGAAGUCCAUCUGAAUU	AGO2 mRNA
siAGO2-1 (antisense)		pUUCAGAUGGACUUCCGUGCUU	
siAGO2-2 (sense)		GCAGGACAAAGAUGUAUUAUU	
siAGO2-2 (antisense)		pUAAUACAUCUUUGUCCUGCUU	
siAGO2-3 (sense)		GGGUCUGUGGUGAUAAAUAUU	
siAGO2-3 (antisense)		pUAUUUAUCACCACAGACCCUU	
siAGO2-4 (sense)		GUAUGAGAACCCAAUGUCAUU	
siAGO2-4 (antisense)		pUGACAUUGGGUUCUCAUACUU	
siTRBP-1 (sense)	siTRBP	AGCCCACCAGCCUAAUUUCdTdT	TRBP mRNA Isoform a and b
siTRBP-1 (antisense)		GAAAUUAGGCUGGUGGGCUdTdT	
siTRBP-2 (sense)	siTRBP-b	GCGGGCUGCCUAGUAUAGAdTdT	TRBP mRNA Isoform b
siTRBP-2 (antisense)		UCUAUACUAGGCAGCCCGCdTdT	

siRNA sequences are listed 5' to 3'. The oligomers have two dT or U overhangs at their 3' end. siAGO1-b was used as a pool of 2 different siAGO1 at total 25 nM (siAGO1-2 (12.5 nM) + siAGO1-3 (12.5 nM)). siAGO2 was used as a pool of 4 different siAGO2 at total 25 nM (siAGO2-1 (6.25 nM) + siAGO2-2 (6.25 nM) + siAGO2-3 (6.25 nM) + siAGO2-4 (6.25 nM)).
